# Reference range variation in haematological indices amongst five different age groups of less than one year in Islamabad, Pakistan

**DOI:** 10.12669/pjms.292.3067

**Published:** 2013-04

**Authors:** Kiran Tauseef Bukhari, Humaira Zafar

**Affiliations:** 1 Dr. Kiran Tauseef Bukhari, MBBS, M.Phil-Haematology, Assistant Professor of Haematology, Al Nafees Medical College, Islamabad, Pakistan.; 2 Dr. Humaira Zafar, MBBS, M.Phil-Microbiology, Assistant Professor of Microbiology, Al Nafees Medical College, Islamabad, Pakistan.

**Keywords:** Haematological Indices, Reference Values, Infants

## Abstract

***Objective: ***The objective of the current study was to establish the reference ranges of haematological indices amongst five healthy infantile (<1 year) age groups.

***Methodology: ***It was a descriptive cross sectional study carried out at the Department of Haematology, Armed Forces Institute of Pathology (AFIP), Rawalpindi. Non probability convenience sampling was adopted for the proceedings for the study. A sample size was 2000 which was equally distributed as 400 samples for all the five age groups i.e. <27 days, 3 month, 6 month, 9 month and one year. One thousands were males and 1000 were the females. An informed consent from the guardian was the pre requisite of study, while those candidates having an evidence of any systemic illness were not excluded.

***Results*** *: *The values of haematological indices i.e. MCV, MCHC, MCH, PCV and RDW varies with growing age of an infant. A decrease in all these values was observed from <27days to one year of age infants.

***Conclusion*** **:** A decrease in all these values was observed from <27days to one year of age infants. The values reported in this study can be used as a local reference for the newborn aged between <27 days and 1 year of age.

## INTRODUCTION

As per IFCC, the reference values are the clinical measurements which are interpreted against the values extracted from the control subjects/groups in order to exactly interpret the health status.^1^^,^^2^ The main concept of establishing the reference values had been adopted by the health care professionals, chemists, laboratory staff, and clinicians in view of establishing the legislations.^3^

The literature review of different areas of world has shown a great variation in haematological parameter and indices due to various factors involvement like socioeconomic status, maternal age/ mode of delivery, complications during delivery, sampling site, high altitude, seasonal variation etc.^4^^-^^6^ The accuracy of reference values is important in order to diagnose and manage a disease prior the occurrence of its complications.^7^^-^^9^

The National literature is very deficient on the subject. However, only one published study results are available which was carried out at Karachi, Pakistan by Qaisar et al; (2009). The study had focused the reference values of mean haemoglobin, total white blood cell counts (WBCs) and plateletcounts.^10^

Therefore this study was planned to establish the reference values of haematological indices amongst various infantile age groups. The accurate comparison will help to early diagnose and hence mange the disease.

## METHODOLOGY

The study population included the samples from infants visiting the vaccination centers and pediatric outpatient departments (OPD’s) of Military Hospital, Combined Military Hospital, Holy Family Hospital, and Benazir Bhutto Hospital, Rawalpindi. Permission to carry out the study was taken from the Ethical Review Committee of Armed Forces Institute of Pathology (AFIP), Rawalpindi.

The sample size was 2000, blood samples with an equal gender distribution i.e. 400 (200 male + 200 female) for all five groups i.e. <27 days(Group I), 03 month(Group II), 06 month(Group III), 09 month (Group IV) and 01 year of age(Group V) were included in the study.

It was a descriptive cross sectional study carried out at Department of Haematology, Armed Forces Institute of Pathology (AFIP), Rawalpindi from 25th March 2010 to 25th March 2011 bynon-probability convenience sampling method. 

 After a brief clinical examination healthy, conscious and a febrile neonates to one year of age group infants were included in the study. While pre mature infants, or those having a history of any congenital disorder, blood loss, use of drugs, haematenics, presence of systemic illness or in case infant's guardian not willing for consent, all were excluded from the study.

A written consent from parents was the pre-requisite of study. Afterwards 2.5 ml of venous blood sample was drawn aseptically from all the candidates and preserved in Ethylenediamine tetra acetic acid (ED'TA) anticoagulant. These were ultimately analyzed by Sysmex KX-21 machine (three part differential) at Haematology Department of AFIP for blood complete picture indices [MCV, MCH, MCHC, Hematocrit (HCT) and RDW. Analysis of variance, frequencies, percentages, mean values and standard deviations were assessed for statistical inference by SPSS version 16.P value less than 0.05 was considered significant different.

## RESULTS

Numerical variables were age, MCV, MCH, MCHC, PCV and RDW. While gender was considered as a categorical variable. Various grouping of numerical values was formulated and respective percentages were measured. Mann-Whitney test was employed to compare all the quantitative variables for male and female within each group. 

A significant difference (p Value <0.05) was noted for HCT when compared the values of newborn-03 month, newborn-06 month, newborn-09 month, and newborn-one year, 03 month-06 month, 03 month-09 month, 03 month-1 year of age. While the difference was non-significant between 06 month-09 month, 06 month-1 year and 9 month-1 year. 

A significant difference (p Value <0.05) was noted for MCV when compared the values of newborn- to remain four groups, 03 month to remaining three groups and 06 months to remaining two groups. While non-significant difference was observed between 09 months and 01 year of age infants.

A significant difference (p Value <0.05) was noted for MCH when compared the values of newborn- to remain four groups, 03 month to remaining three groups and 06 months to remaining two groups. While non-significant difference was observed between 09 months and 01 year of age infants.

A significant difference (p Value <0.05) was noted for MCHC when compared the values of newborn- to remain four groups, 03 month to remaining three groups, 06 months to remaining two groups and 09 months to 01 year of age infants.

A significant difference (p Value <0.05) was noted for RDW when compared the values of newborn- to remain four groups, 03 month to 06 months, and 06 months to 01 year & 09 months to 01 year. While non-significant difference was observed between 03 months to 09 months, 03 to 01 year and 06 to 09 months of age infants.

Amongst the quantitative variables, mean values and the standard deviation were used in order to assess the accuracy of results amongst males and females of different age groups (< 27 days to 1 year) as shown in Table-I.

**Table-I:** Data of the haematological indices in different infantile age groups

*Sr.* *No*	*Haematological Parameter*	*< 27 DAYS* *Mean ± SD*	*3 MONTH Mean ± SD*	*6 MONTH Mean ± SD*	*9 MONTH Mean ± SD*	*1 YEAR Mean ± SD*
1.	Hct l/l	0.50(0.06)	0.31(0.04)	0.32(0.03)	0.33(0.03)	0.32(0.03)
2.	MCV fl	102.31(6.95)	81.36(7.87)	76.23(7.05)	71.54(6.24)	71.25(9.96)
3.	MCH pg	34.37(1.97)	27.12 (3.86)	24.62(2.48)	22.61(2.43)	22.25(3.43)
4.	MCHC g/l	33.62(2.81)	33.13(2.08)	32.33(1.62)	31.57(2.01)	31.08(2.14)
5.	RDW(fl)	66.05 (7.32)	42.47(5.61)	40.76(4.59)	41.48(4.29)	43.39(7.54)

The frequencies and percentages of haematological indices amongst different age groups (< 27 days to 1 year) were the qualitative variables used for the current study as shown in Table-I.

## DISCUSSION

The values of haematological parameters currently used in our country are usually derived from the data of Western populations.^11^^,^^12^

The overall mean MCV found in the current study was lower in < 27 year, 9 month 1 year, comparable to 6 month and higher in 3 month as compared to that in Western population.^12^ Females presented higher value of MCV as compared to male in one year age group. However a decrease in this value was observed with growing age from < 27 days to one year age group. Findings by Myhre et al; (1970) support the current findings. He described a lower MCV value along with high haemoglobin and RBC count as a result of increased erythropoiesis due to hypoxic stimulus at high altitude.^13^

The overall mean MCH value in the study was slightly higher in case of <27 years of age comparable to the value in three months and lower than six months, nine months and one year as compared to the range in Western population.^12^ In this study, the mean values were increased with growing age. Gender does not have effect on the current values of MCH, except in one year age group, where the value was higher in female.

As compared to the Western study, MCHC mean values were lower than that observed in 6 month, 9 month and one year age groups. It was comparable to < 27 days and 3 month age groups. The present mean value decreased with growing age. Like the MCV and MCH, the MCHC mean values in the current study were similar to the observations by Mitchell et al (2006) in infants.^12^

The overall mean value of haematocrit in the current study was higher as compared to the range in Western countries in age group of < 27 days. While the comparable values were observed for 3, 6, 9 months, and one year of age groups.^12^ The current study results have shown that the value was statistically higher in one year old females. This can be comparable with the study carried out at Uganda by Lugada et al in (2004). After a study in Uganda he reported rise in haematocrit with age and no obvious gender effect up to 13 years. Furthermore, variations in haematological reference values between African and Western population has been also reported.^14^

The current mean RDW value was higher in case of < 27 year of age and one year age groups when compared to that in Western populations.^12^ The value was comparable to the reported Western value in 3 month group, lower in 6 month group and 9 month. The RDW mean value of current study in all five age groups have shown higher values i.e. >40% when compared to < 33% reported by Lugada et al (2004) in one year healthy babies.^14^

Choi et al (1998) had described the demographic effect upon variation in different blood counts amongst the residents of Italy, Pakistan Malaysia, New Delhi, Russia and Nigerian.^15^ The reference range must be reviewed for the specific localities due to significant differences and variations.^15^^,^^16^ Pakistan has a strong social setup, rising from the previous century, which leads to certain food habits and food fads that cannot be altered. The huge joint family system with one earning hand results in nutritional deficiencies especially among the growing children. The government is also not able to provide assistance to support such families and individuals.

In Pakistan and other developing countries most of the hospital laboratories are using the reference values and ranges recommended by the Western studies. There are no elaborate studies available locally which could help in formulating reference ranges for haematological indices. The goal of the present study was to establish the reference values for haematological indices in infants of Rawalpindi / Islamabad. This will help the clinicians to compare the laboratory test results with locally generated reference ranges.^12^ They are the useful tools to accurately diagnose and hence manage the underlying disease. Moreover, timely provision of treatment can reduce the infant morbidity and mortality rates.^17^

## CONCLUSION

A variation and decrease in all these reference indices hematological values was observed from <27days to one year of age infants. Therefore the values reported in this study can be used as a local reference for the newborn aged between <27 days and one year of age. These reference values must be reviewed for different localities (due to their dependency upon various demographic factors), in order to implement the accurate ones for comparison.

## Author Contribution

Kiran Tauseef Bukhari: Synopsis writing, sample collection, sample processing, data transfer on SPP, data analysis and compiling the results.

Humaira Zafar: Statistical analysis, compiling the result and drafting/formatting the manuscript.
